# 非小细胞肺癌三级淋巴结构评估及临床应用中国专家共识（2025版）

**DOI:** 10.3779/j.issn.1009-3419.2025.102.03

**Published:** 2025-03-21

**Authors:** 中国抗癌协会肿瘤病理专业委员会肺癌协作组, 中国临床肿瘤学会非小细胞肺癌专业委员会

**Keywords:** 肺肿瘤, 三级淋巴结构, 评估, 临床应用, 共识, Lung neoplasms, Tertiary lymphoid structure, Assessment, Clinical application, Consensus

## Abstract

三级淋巴结构（tertiary lymphoid structure, TLS）在肿瘤微环境中发挥着至关重要的作用，影响肿瘤的发生发展。作为预测肿瘤患者预后及治疗反应的一种新兴生物标志物，TLS越来越受到重视。但目前对于TLS的评估缺乏统一的标准，且TLS在不同肿瘤组织中存在差异，为该标志物在临床转化中的应用带来困扰。为满足非小细胞肺癌（non-small cell lung cancer, NSCLC）临床诊治需求，本共识围绕NSCLC TLS的定义、临床意义、检测内容及评估方法等基本要点，结合相关研究和中国临床实践，为临床TLS评估及应用提供标准化和规范化建议，以提高临床医师及病理检测诊断人员对TLS的认识，为NSCLC TLS检测临床应用提供参考依据。

## Abstract (en)

The tertiary lymphoid structure (TLS) plays a crucial role in the tumor microenvironment, influencing tumor development and progression. As an emerging biomarker for predicting the prognosis and treatment response in cancer patients, TLS has received increasing attention. However, there is currently a lack of standardized evaluation criteria for TLS, and significant differences exist in TLS across different tumor tissues. This poses challenges for the clinical application of this biomarker in translation. To meet the clinical diagnosis and treatment needs of non-small cell lung cancer (NSCLC), this consensus focuses on the definition, clinical significance, testing components, and assessment methods of TLS in NSCLC. Combining relevant research and Chinese clinical practice, it provides standardized and normalized suggestions for the clinical assessment and application of TLS, so as to improve the understanding of TLS among clinicians and pathologists, and provide a reference basis for the clinical application of the detection of TLS in NSCLC.

对于非小细胞肺癌（non-small cell lung cancer, NSCLC）免疫检查点抑制剂（immune checkpoint inhibitors, ICIs）为主的免疫治疗，程序性细胞死亡配体1（programmed cell death ligand 1, PD-L1）表达是目前应用最广的肿瘤免疫治疗生物标志物^[1]^，但是部分阴性患者可以从免疫治疗中获益，部分阳性患者未能获益。因此，正确认知PD-L1的不完美性，挖掘具有更高预测效能的生物标志物来筛选免疫治疗的获益者，已经成为目前临床研究的热点。近年来，三级淋巴结构（tertiary lymphoid structure, TLS）与肿瘤预后或免疫治疗关系的研究报道越来越多，多项研究^[2,3]^显示，TLS与肿瘤ICIs疗效有较高的相关性。无论PD-L1表达状态如何，肿瘤内TLS与患者的临床获益呈正相关。NSCLC相关研究^[2,4]^表明，TLS的存在与NSCLC良好预后和免疫治疗获益相关，有望成为预测NSCLC免疫治疗疗效的生物标志物。但在临床研究和实践过程中TLS评估尚缺乏统一标准。

为统一TLS检测和临床应用的认识及促进TLS评估的标准化，中国抗癌协会肿瘤病理专业委员会肺癌协作组与中国临床肿瘤学会非小细胞肺癌专业委员会共同组织国内多领域、多学科专家，综合国内外有关TLS研究的重要文献及临床实践，编写《非小细胞肺癌三级淋巴结构评估及临床应用中国专家共识（2025版）》（以下简称“共识”）。本共识针对NSCLC TLS检测的定义、临床意义、检测内容、所涉及的标志物、评估标准化等问题达成共识并给出指导性建议，以期帮助并推动TLS在NSCLC临床诊治中的标准化和规范化。

## 1 方法学

共识的制定主要基于近些年TLS相关的临床研究和文献，专家组成员梳理临床问题进行充分讨论，针对关键问题进行投票，汇总专家意见及投票结果，根据专家组投票的一致性程度确定推荐级别（表1），最终形成共识推荐内容。

**表1:** 共识推荐级别分类

推荐级别		专家组共识强度
强烈推荐		专家组一致共识（投“非常同意”的票数超过2/3）
一般推荐	I级	专家组基本一致共识（投“非常同意”+“基本同意”的票数超过2/3）
	II级	专家组无共识（投“非常同意”+“基本同意”的票数低于2/3）
不推荐		专家组无共识（投“不同意”的票数高于2/3）

## 2 TLS定义及评估的临床意义

### 2.1 TLS定义与分类

相较于初级淋巴器官/组织（胸腺和骨髓）和次级淋巴器官（secondary lymphoid organs, SLO）/组织（脾脏、淋巴结和扁桃体等），TLS是非生理条件下非淋巴组织中继发产生的免疫细胞聚集体，与淋巴组织具有相似的结构和功能^[5]^，一般出现于自身免疫性疾病、慢性感染、癌症所致的慢性炎症病变中^[6]^。

TLS的成熟依赖于先天性免疫细胞和适应性免疫细胞^[7]^，与SLO类似，主要以通过高内皮微静脉（high endothelial venule, HEV）渗出的滤泡树突状细胞（follicular dendritic cell, FDC）和成熟B细胞的增多为特征^[8]^，成熟的TLS往往提示肿瘤患者有良好的预后。根据不同的研究目的，TLS的分类方法也不尽相同。

TLS的成熟可细分为3个阶段^[9⇓⇓⇓⇓⇓⇓-16]^。早期TLS（early TLS, E-TLS）主要由密集的淋巴细胞聚集组成，缺乏CD21和CD23信号，没有FDC，也没有隔离的T细胞区和B细胞区，存在或不存在散在的CD4^+ ^T细胞；原发性滤泡样TLS（primary follicle-like-TLS, PFL-TLS），又名初级TLS，存在未成熟的FDC，但未观察到生发中心（germinal center, GC），具有CD21但没有CD23信号；继发性滤泡样TLS（secondary follicle-like-TLS, SFL-TLS），又名次级TLS，具有CD21和CD23信号的致密淋巴细胞聚集体，存在活跃的GC反应，类似于SLO。继发性滤泡样TLS又被称为成熟的TLS。

TLS可分为未成熟和成熟TLS^[14,17,18]^。成熟TLS有T细胞、B细胞淋巴样聚集、GC和FDC形成，表现为CD21^+^和CD23^+^。未成熟TLS虽然有T细胞和B细胞淋巴样聚集，但没有GC，也没有FDC的存在。

TLS可分为阳性和阴性两类^[19,20]^。TLS阳性（TLS^+^）定义为肿瘤及肿瘤浸润边缘（infiltration margin, IM）内可见淋巴细胞聚集体，淋巴细胞数量>50个^[21]^，相反则定义为TLS阴性（TLS^-^）。

其他关于TLS的分类：比如将TLS分为肿瘤内TLS（intra-TLS）和肿瘤周围TLS（peri-TLS），或者根据GC是否存在，还可将TLS分为GC^+ ^TLS、GC^- ^TLS^[9,22,23]^。

**共识一：TLS是非生理条件下非淋巴组织中继发产生的免疫细胞聚集体（至少由50个细胞组成），与淋巴组织具有相似的结构和功能。TLS按照不同的研究目的有不同的分类，依据是否存在GC和FDC，大致可分为成熟TLS与未成熟TLS。**

**证据级别：强烈推荐。**

### 2.2 TLS的临床意义

#### 2.2.1 TLS预后价值

一般来说，未成熟TLS主要与免疫抑制的肿瘤微环境（tumor microenvironment, TME）相关，很少诱导有效免疫应答^[24]^，而成熟TLS可有效激活机体的体液免疫^[25⇓-27]^。研究^[28,29]^表明不同类型实体肿瘤样本中高浸润成熟TLS，患者预后良好，TLS的预后预测价值通常与肿瘤原发灶-淋巴结-转移（tumor-node-metastasis, TNM）分期无关，但与TLS密度、成熟度、组成等因素相关。

2008年有研究^[30]^首次报道了NSCLC中TLS相关数据，提示TLS结构中成熟的树突状细胞（dendritic cell, DC）数量可以预测早期NSCLC患者的高复发风险。一项研究^[31]^通过免疫组织化学（immunohistochemistry, IHC）评估538例未接受治疗的NSCLC患者肿瘤样本中的TLS状态，发现TLS内的B细胞（TLS-B）高密度能够抵消高Treg密度对患者生存的有害影响，TLS-B^high^Treg^low^患者可获得更好的临床结局。在111例NSCLC患者接受新辅助化疗（放疗）后手术切除的样本中发现TLS数量越多，CD8^+^肿瘤浸润淋巴细胞（tumor infiltrating lymphocytes, TILs）密度越高（P=0.009），与较长的总生存期（overall survival, OS）（P=0.014）和更长的无病生存期（disease-free survival, DFS）（P=0.008）相关^[32]^。TLS内程序性细胞死亡受体1（programmed cell death 1, PD-1）的功能失调已被确定为晚期NSCLC患者PD-1抑制剂响应的预后指标。TLS丰度与免疫细胞PD-L1表达相关，而与肿瘤细胞PD-L1表达无关^[33]^。还有研究^[34]^表明，新辅助化学免疫治疗后病理学完全缓解（pathological complete response, pCR）的NSCLC患者，其DFS的延长与TLS的成熟度和更高的TLS密度相关。

#### 2.2.2 TLS的疗效预测价值

TLS作为肿瘤内局部免疫激活的标志，其存在往往与患者对ICIs（如PD-1/PD-L1抑制剂）等免疫疗法的更好响应相关，如肝癌^[10]^、乳腺癌^[35]^、胰腺癌^[36]^等。TLS丰富的免疫细胞群和免疫激活环境可能增强了对免疫治疗的敏感性。因此，TLS指标检测有助于筛选出可能从免疫治疗中获益的患者群体。

通过对接受抗PD-1/PD-L1治疗的泛癌种（包括127例的NSCLC）患者三个独立队列（n=328）大规模回顾性分析，发现成熟TLS的存在与客观缓解率（objective response rate, ORR）、无进展生存期（progression-free survival, PFS）和OS的全面提高相关，与PD-L1表达状态和CD8^+ ^T细胞密度无关^[3]^。接受新辅助化疗联合免疫治疗的NSCLC研究^[37,38]^发现，TLS在有响应的患者中更容易观测到，且可显著改善主要病理缓解（major pathologic response, MPR）组的DFS（P=0.019）。另一项接受新辅助化疗的NSCLC的研究^[39]^中也显示MPR组患者有更高密度的TLS，TLS密度越高（阈值=0.5550个/mm^2^），患者接受新辅助化疗的疗效越好。

**共识二：TLS与NSCLC患者的预后和疗效密切相关，肿瘤内TLS密度越高、成熟度越高，患者的获益越明显。**

**证据级别：强烈推荐。**

## 3 TLS的评估内容

### 3.1 TLS的成熟度与密度 TLS的成熟度详见“2.1 TLS定义与分类”。

TLS密度计算为肿瘤周围和肿瘤内区域每平方毫米TLS的个数^[4,40⇓-42]^。这种基本的定量方法适用于所有类型的肿瘤研究，具有操作性和稳定性^[43]^。在肺癌的不同队列研究^[44⇓-46]^中，TLS的密度主要是依据TLS的个数与切片面积的比值来计算。有研究^[26]^通过TLS密度的中位数将患者群体分为TLS^high^组和TLS^low^组。一项研究^[47]^纳入616例NSCLC患者样本，10×镜下对瘤内淋巴细胞聚集体的TLS密度进行了检查，并将0.1 TLS/mm^2^用作TLS^low^和TLS^high^肿瘤分类的阈值。另一项研究^[48]^在探讨肺腺癌TLS密度的临床意义中，研究者将TLS密度的阈值确定为0.489 TLS/mm^2^。

**共识三：评估TLS，需要关注TLS的成熟度和密度，TLS成熟度的定义参考共识一，TLS的密度可定义为肿瘤周围和肿瘤内区域每平方毫米TLS的个数，一般利用中位数区分TLS的高密度和低密度。**

**证据级别：一般推荐I级。**

### 3.2 TLS的位置

TLS在肿瘤内、IM和肿瘤周围中均有分布，位于肿瘤不同位置的TLS对预后评估的作用也可能不同。目前大多并无细致区分TLS的具体定位或探究TLS不同定位与肿瘤诊疗或预后之间的联系，即缺乏明确的浸润位置的判读标准。

有研究^[49]^将TLS定义为位于肿瘤内或距IM最多1 mm内的由CD20^+^和CD3^+^细胞（由至少50个细胞组成）组成的细胞聚集体。有关乳腺癌的研究^[50]^将IM定义为从侵入边界延伸至1 mm的基质区域，《CSCO乳腺癌诊疗指南2024》明确将IM定义为以机体正常组织与癌巢分界为中心1 mm的范围区域^[51]^。而关于IM的定义也有研究^[11]^界定在肿瘤两侧宽度为500 μm以内，所以若TLS的位置在IM以外，可将TLS判读为肿瘤周围的TLS。还有研究^[32]^将TLS定义为肿瘤内和肿瘤边缘7 mm范围内的所有淋巴细胞聚集体，以及通过肿瘤近端或肿瘤远端来定义TLS的位置，肿瘤近端TLS被定义为位于IM内的TLS，而肿瘤远端TLS被定义为位于距离IM>10 mm的正常组织内的TLS^[42,52]^。另外，还可以根据PD-L1染色综合阳性评分（combined positive score, CPS）判读时所参照的20倍原则对TLS进行定位，即在20倍放大视野内，将肿瘤边缘或肿瘤病灶置于视野中央，即可定位TLS的相对位置。

**共识四：TLS在肿瘤中的位置可分为肿瘤内、IM和肿瘤周围，后两者的划分目前大致推荐以IM 1 mm内为参考数值。TLS的阳性评估主要关注肿瘤内和IM。**

**证据级别：一般推荐I级。**

## 4 TLS评估所涉及的相关标志物

TLS的细胞组成和结构在一定程度上决定了其功能。TLS主要由T细胞、B细胞、FDC、DC、基质细胞、巨噬细胞、内皮细胞和HEV组成。TLS在细胞水平上包括三个主要组成部分：外部T细胞区、内部B细胞区和周围的HEVs^[53]^。T细胞区包含CD4^+ ^T细胞簇、CD8^+ ^T细胞簇、DC-LAMP^+^或CD83^+^的成熟DC簇^[5,53,54]^。GC发育成含有表达CD21/CD23的FDC和若干B细胞的B细胞区，这些细胞还包括效应B细胞、浆细胞和记忆B细胞，TLS的大多数抗肿瘤作用都与之相关^[5,43,55]^。此外，TLS中的B细胞可能在ICIs治疗后参与有效的T细胞反应^[43]^。

根据不同临床研究所涉及的研究指标各不相同，最常用的TLS检测指标包括：T细胞（CD3/CD8/CD4）、B细胞（CD20）、FDC（CD21/CD23/DC-LAMP）及其他细胞（表2^[3,5,49,56⇓⇓⇓⇓⇓⇓⇓⇓⇓⇓⇓⇓⇓⇓-71]^）。在评估患者预后时，有CD23表达的成熟TLS可以显著改善患者预后^[3,42]^。在检测过程中，若发现TLS中有较高比例的FoxP3^+^Treg细胞，则会提示免疫应答能力减弱^[72]^。而在评估免疫联合抗血管生成治疗NSCLC的疗效时，在常规检测TLS的同时，加上HEVs（PNAd）的检测，结果显示PNAd^+^的TLS可有效预测免疫治疗联合抗血管生成治疗NSCLC的疗效^[73]^。

**表2:** 常见的TLS标记指标

指标	细胞/组织类型	检测指标	参考文献
主要指标	T细胞	CD8、CD3、CD4	^[3,5,49,56⇓⇓⇓⇓⇓⇓⇓-64]^
	B细胞	CD20	^[3,5,49,56⇓⇓⇓⇓⇓⇓-63,65]^
	滤泡树突状细胞	CD23、CD21	^[3,59,60,62,63,66,67]^
次要指标	肿瘤细胞	panCK	^[64,68,69]^
	其他	PNAd、FoxP3、CD19、DC-LAMP	^[47,49,54,57,61,63⇓-65,70,71]^

TLS: 三级淋巴结构

**共识五：TLS不同成熟阶段，其细胞组成不同，检测TLS至少要纳入CD8（T细胞）、CD20（B细胞）、CD23（FDC）这三个指标，根据不同的研究目标，可针对性地纳入panCK、CD3、CD4、CD21、PNAd、FoxP3、CD19、DC-LAMP等指标更好地分析TLS中细胞的组成和作用，建议选定6-7个合适的指标来检测TLS。**

**证据级别：强烈推荐。**

## 5 TLS评估

### 5.1 TLS检测的样本类型和质控

目前研究涉及的TLS检测样本主要有手术切除样本、穿刺活检样本以及不同组织类型的原发灶、复发灶、转移灶和治疗前后的病灶^[21]^，其中手术切除样本约占80%。活检样本和手术样本均能检测到1至多个成熟的TLS^[3]^，控制好其他变量后检测结果可以达到良好的一致性^[74]^。

考虑到样本的可及性和细胞数量等因素，优先推荐10%中性缓冲福尔马林固定石蜡包埋（formalin-fixed paraffin-embedding, FFPE）的手术切除样本进行TLS检测，需要排除含有大量坏死组织、挤压细胞及固定不佳的样本^[21]^。在没有手术切除样本的情况下，可以用活检样本开展检测，应尽可能检测所有同批次活检组织，合并计算后报告TLS检测结果，并备注小样本存在低估或者漏检TLS的情况。活检样本应确保取材位置的准确性，并有足够的肿瘤细胞进行评估。研究^[75,76]^表明活检样本的大小与TLS结果准确性呈正相关（P<0.001）。由于肿瘤的异质性以及TLS出现的位置不固定，活检样本无法代表整个肿瘤的实际表达状态，有可能存在TLS被低估或者漏检情况。

TLS检测前，需要对苏木素-伊红（hematoxylin-eosin, HE）染色的切片进行病理评估，观察是否存在脱片、皱褶、未染色等现象，评估HE切片中红细胞和坏死细胞比例，红细胞、坏死细胞比例应控制在20%以内。参照免疫组化（immunohistochemistry, IHC）检测技术共识，TLS评估结果受检测前后多种因素的影响，应用于临床检测前，各医疗机构应建立标准化操作流程，完成性能验证，验证参数包括特异度、灵敏度、稳定性和重现性^[1]^。

**共识六：用于TLS评估的样本可以是手术或者活检样本，优先推荐手术样本，若只有活检小样本，可以根据样本质控结果酌情考虑是否开展TLS评估。样本处理应严格按照科室相关标准化操作程序（Standard Operating Procedure, SOP）文件的要求进行。**

**证据级别：强烈推荐。**

### 5.2 TLS评估方法和评估标准

目前检测TLS的常规方法包括HE、IHC、免疫荧光（immunofluorescence, IF）和多重荧光免疫组化（multiplex immunohistochemistry/immunofluorescence, mIHC/mIF）等^[21,77]^。

#### 5.2.1 HE

对肿瘤组织切片HE染色是检测TLS最简单的方法。通过HE染色可以观察到TLS的大体形态和组织结构，但仅限于待测区域形态表面特性等有限信息的获取，不能分析细胞分子层面的免疫表型等特征指标。HE染色可用于TLS结构的初步判断。

#### 5.2.2 IHC

通过IHC染色法在组织中检测TLS已成为较为普遍的形式，且特异性较好，可鉴定CD4^+ ^T细胞、CD8^+ ^T细胞和CD20^+ ^B细胞以及CD21^+^/CD23^+ ^FDC，是目前评估TLS指标最为简便易行的检测方法。使用针对特定免疫细胞标志物（如CD20、CD3、CD21、CD23、CXCL13、PD-L1等）的单克隆抗体进行染色，通过显色反应在显微镜下观察和量化TLS内相应免疫细胞的分布和数量，以判断TLS成熟程度。但单张切片仅能实现单指标检测，难以准确评估多个指标间的表达关系情况。

#### 5.2.3 IF

使用荧光标记的抗体，能在同一视野下通过不同的荧光信号对多种免疫细胞或分子进行同时标记，提供更高分辨率的空间定位信息，有助于揭示TLS内细胞间相互作用和微环境动态。但是，由于抗体种属的局限性，一般最多标记3种指标，临床实践中仍未得到广泛使用。

#### 5.2.4 mIHC/mIF

类似于IHC染色抗原抗体结合的方式，但不同于DAB显示方式，而是在一张组织切片上通过多个荧光信号来标记不同的待检抗原，再结合计算机辅助图像分析，可以精确量化多种免疫细胞在TLS内的比例和相互关系。传统的病理诊断依赖病理医生对病理切片上的细胞和组织学病变进行阅读和分析，从而确定肿瘤类型^[78]^。mIHC/mIF技术在保证分辨率的同时可兼顾全景成像，凭借视场大、颜色多、通量高的特点，可在短时间内原位检测整张组织切片上的多种生物标志物的表达情况，借以识别组织上每个细胞表型、丰度、状态及其相互关系。在TLS的检测过程中，一般先进行HE染色，选取TLS表达的样本区域，再用mIHC/mIF对TLS进一步解析。但mIHC/mIF技术对平台硬件和分析人员有一定要求，目前在国内尚属于新技术使用转化阶段。

此外，转录组测序等技术可以从分子层面深入解析TLS内细胞异质性、细胞间通讯和功能状态，提供更全面的TLS组成和功能信息。目前此类技术仍局限于临床研究阶段。

关于TLS的病理评估，首先需要确认HE染色切片上是否存在TLS，并结合IHC判断其位置、数量及成熟度。有条件的机构或医学中心可利用mIHC/mIF技术，结合数据分析软件进行定量分析，按照既定的分析流程和算法分别对组织、细胞进行分割，统计各靶标在肿瘤相关区和间质区的表达情况，进行判读分析^[21]^。评估标准：当出现免疫细胞聚集体（多于50个细胞），且CD20（B细胞表达）、CD8（T细胞表达）、CD23（FDC表达）均有表达时，则可判读成熟TLS；若只有CD20、CD8表达，则判读不成熟TLS；若CD20、CD23均未表达，则判读未发现TLS。

**共识七：评估TLS的技术方法包括HE、IHC、IF和mIHC/mIF等，推荐HE结合IHC对TLS的位置、数量和成熟度进行常规评估。推荐有条件的医疗机构，使用mIHC/mIF定量分析TLS的密度、大小和数量，以及TLS中各类TILs的组成及浸润丰度，综合评估TLS是否存在及成熟度。**

**证据级别：强烈推荐。**

### 5.3 评估报告模板

TLS评估结果报告应包括患者基本信息、样本病理信息、使用的技术方法、相关质量控制信息、评估结果及必要的备注。关于TLS的评估内容，应在报告中呈现TLS的成熟度、密度、个数、位置等内容。备注部分包括对样本特殊之处的说明、适当注释检测结果的临床意义等信息。

**共识八：TLS检测报告模板建议涵盖所有的基本信息，并体现TLS评估技术和TLS的成熟度、密度、个数、位置等内容。**

**证据级别：一般推荐I级。**

## 6 尚待解决的问题

TLS作为一种新兴的抗肿瘤免疫相关指标，越来越受到人们的关注。但许多研究细节尚待确定，如TLS在肿瘤中的位置可分为肿瘤内、IM和肿瘤周围，这可能会影响TLS的临床意义与价值^[79]^，但IM和肿瘤周围的准确区分标准尚无定论。另外，肿瘤周围和肿瘤内的TLS似乎具有不同的功能，这种差异定位的驱动因素尚不清楚，不同位置的TLS影响肿瘤免疫的机制有待进一步阐明。

TLS密度的计算也是有待明确的一个问题，大多数研究均将TLS的密度定义为每平方毫米中TLS的个数，需明确知道统计的TLS个数是基于局部的几个视野还是整张切片，如果仅是基于局部的几个视野，这些视野划分的依据又是什么?判断TLS密度高与低的临界值又如何获取等等，这些都有待进一步探索。

TLS评分系统包括两种类型：基于TLS及其相对于肿瘤位置（肿瘤区和肿瘤周围）细分下的分布特征的分级评分^[80,81]^和基于全片TLS分布数量的分级评分^[33,37,82]^。目前NSCLC中TLS的评分系统只有少量的科研文献支撑，未来仍需要更多临床研究验证或完善，以确认TLS的评分系统在NSCLC患者中的临床价值。

作为一种预测NSCLC患者疗效或预后的新型生物标志物，TLS的未来发展关注点主要包括两个方面。一是TLS指标虽已被广泛研究，但目前仍没有统一的TLS评估标准，缺乏多中心、大样本的研究验证并给出详实的数据来阐述TLS在NSCLC患者中的准确诊疗指导作用。二是技术操作和平台使用的稳定性及可及性得到保障，软件分析能满足临床需求。这些需要技术研发人员、临床医师和病理医师的密切合作和共同努力。另外，其他成像技术和人工智能在组织病理学中的联合应用，或可能为TLS评估开辟新的途径。

## 7 总结

TLS作为NSCLC诊疗领域的新兴生物标志物，越来越受到临床医师和病理医师的关注和重视。一系列回顾性队列和临床研究^[28⇓⇓⇓⇓⇓⇓⇓⇓⇓⇓-39]^表明，TLS的空间结构如GC的存在、高密度TILs浸润、免疫细胞表型差异及其在肿瘤区域的分布等会影响肿瘤患者的预后与疗效；多种评估TLS的技术方法以HE结合IHC优先推荐，并以规范检测前样本处理和质控作为评估保障，助推并提高未来TLS作为生物标志进行临床转化的可行性。

## 8 免责声明

协作组撰写的此份共识仅代表协作组的专家共识和经验。本共识中的推荐不应被视为完整或绝对正确，也不应被视为一成不变的临床实践准则，更不能替代临床专科医生的独立判断。随着循证医学的快速发展，新证据可能会在本共识撰写、修改、杂志审校排版发表期间出现，本共识中所引用的研究（2024年10月前收录于PubMed的相关文献）可能无法反映最新循证。
